# Soil-Temperature-Compensated Growing Degree Days Improve Unified Simulation of Maize LAI Dynamics Across Film Mulching Treatments

**DOI:** 10.3390/plants15142163

**Published:** 2026-07-14

**Authors:** Wangwang Zhang, Yuanzheng Zhang, Weishu Wang, Shijun Sun

**Affiliations:** 1 College of Water Conservancy, Shenyang Agricultural University, Shenyang 110866, China; 2021200011@stu.syau.edu.cn (W.Z.); wangws@syau.edu.cn (W.W.); 2 Nanjing Institute of Environmental Sciences, Ministry of Ecology and Environment of China, Nanjing 210042, China; zhangyuanzheng@nies.org

**Keywords:** canopy development, growing degree days, compensation effect, Logistic growth model

## Abstract

Film mulching can promote maize canopy development by altering soil thermal conditions. However, commonly used air-temperature-based growing degree days (GDDs_air_) may not adequately reflect mulch-induced soil warming or the effects of biodegradable film degradation on leaf area index (LAI) dynamics. To improve unified simulation of maize LAI under different film mulching conditions, field experiments were conducted in 2023 and 2024. Five treatments were established: 0.006, 0.008 and 0.010 mm biodegradable films (DM1, DM2 and DM3, respectively), a 0.010 mm conventional plastic film (PM), and a no-mulching control (CK). The compensation of increased soil temperature for air-temperature-based thermal accumulation during early maize growth was quantified. Modified Logistic LAI models were then developed using days after emergence (DAEs), GDDs_air_, soil-temperature-compensated growing degree days (GDDs_stc_), and normalized GDDs_stc_ (NGDDs_stc_) as driving variables. The models were calibrated with observations from 2023 and independently validated with observations from 2024. The compensation effect acted through mulch-induced increases in 0–10 cm soil temperature during early maize growth and was stronger at the seedling stage than at the jointing stage. Compared with DM1 and DM2, daily compensation values were higher by 0.25–0.78 °C under DM3 and by 0.26–0.76 °C under PM. Independent validation showed that the GDDs_stc_-driven model had lower prediction error than the DAEs- and GDDs_air_-driven models. The NGDDs_stc_-driven model performed best; its RMSE values were 17.61%, 15.17% and 10.91% lower than those of the DAEs-, GDDs_air_- and GDDs_stc_-driven models, respectively. These results indicate that incorporating mulch-induced soil temperature compensation into the thermal time scale can more accurately represent maize canopy development under film mulching conditions.

## 1. Introduction

As one of the most widely cultivated cereal crops worldwide, maize plays a key role in sustaining food security and feed supply [1]. Leaf area index (LAI) is a key indicator of maize canopy structure, light interception capacity and photosynthetic potential. It reflects canopy growth dynamics and strongly influences photosynthate accumulation and subsequent yield formation [2]. During the maize growing season, LAI typically increases rapidly, reaches a peak, and then declines as leaves senesce, yellow and abscise. LAI dynamics are jointly regulated by genotype, planting density, water availability, nutrient status, light and temperature [3,4,5]. From a physiological and ecological perspective, leaf growth rate is highly dependent on the external light and thermal environment, in addition to genetic control. LAI is therefore often regarded as a key intermediate variable linking soil hydrothermal processes, canopy energy exchange and crop growth simulation. Under conditions without severe water or nutrient stress, temperature is generally considered to play a dominant role in LAI dynamics [6]. Air-temperature-based growing degree days (GDDs_air_) convert chronological time, such as days after emergence (DAEs), into a thermal time scale and are therefore widely used in crop models such as DSSAT, APSIM and AquaCrop to describe crop phenology and canopy growth dynamics [7,8].

Film mulching is a widely used agronomic practice in maize production in arid, semi-arid and cool regions [9,10]. However, conventional polyethylene film (PE) degrades slowly under field conditions, and its long-term, large-scale use has caused serious residual plastic film pollution in croplands, which is detrimental to agricultural sustainability [11]. Biodegradable mulch films are considered a promising alternative to PE films [12,13]. Numerous studies have shown that biodegradable mulch films can provide soil-warming and moisture-conserving effects similar to those of PE films [14,15]. However, these mulching effects are often dynamic because they are influenced by intrinsic film properties, such as material composition, thickness and degradation rate, as well as environmental factors, including radiation, wind, temperature and precipitation [16]. The resulting variation in soil hydrothermal conditions further affects maize growth, development and yield formation [17,18].

Crops require a relatively stable amount of accumulated heat to complete a given developmental stage in the absence of severe stress [19,20]. However, under film mulching, GDDs_air_ calculated only from air temperature may not adequately capture the actual dynamics of maize canopy development [21]. At the same GDDs_air_, mulched maize may show a faster rate of LAI expansion. This phenomenon can be explained by the increase in soil temperature under film mulching. The additional soil heat accumulation can partly compensate for the air-temperature-based thermal requirement needed to complete a given developmental stage. As a result, LAI development is advanced under mulched conditions [22]. Meanwhile, the soil-warming effect of biodegradable mulch films decreases as films degrade, making their effects on LAI development more complex than those of PE films [23]. Therefore, incorporating the compensation effect induced by film mulching into GDDs_air_ calculation is necessary to more accurately describe LAI dynamics under different mulching conditions [24]. Previous studies have attempted to incorporate the compensatory effect of film mulching into crop growth modelling. Zou et al. [21] quantified the compensatory effect of plastic film mulching on GDDs_air_ in a wheat–maize rotation system and used a Logistic equation to describe plant height dynamics. Zhang et al. [22] further integrated the compensatory effect into the AquaCrop model to improve maize growth and development simulation under plastic film mulching.

The Logistic growth model has clear biological interpretability and requires relatively few parameters. It has therefore been widely used to simulate crop growth processes [25]. The classical three-parameter Logistic model is suitable for describing processes that increase monotonically and gradually approach a plateau, such as plant height and dry matter accumulation. However, it cannot adequately represent the decline in LAI during late growth stages [26,27]. A modified Logistic model is therefore needed to describe the unimodal pattern of LAI dynamics over the maize growing season. In addition, most existing LAI simulation models use GDDs_air_ as the driving variable and insufficiently account for the soil-warming effect induced by film mulching. They are even less able to represent the weakening of this warming effect as biodegradable films degrade. The resulting mismatch in canopy developmental timing among treatments limits the accuracy of unified LAI simulation models. Based on this background, this study aimed to: (1) quantify the compensation effect under biodegradable and PE film mulching; and (2) develop modified Logistic LAI models driven by DAEs, GDDs_air_, soil-temperature-compensated growing degree days (GDDs_stc_), and normalized GDDs_stc_ (NGDDs_stc_). We hypothesized that incorporating mulch-induced soil temperature compensation into GDDs_air_ would provide a more effective thermal time scale for unified LAI simulation than DAEs or GDDs_air_ alone, and that segmental normalization of GDDs_stc_ would further help reduce interannual and treatment-specific differences in LAI development.

## 2. Results and Discussion

### 2.1. Compensation Effect Under Film Mulching

Different mulching treatments showed clear variation in the compensation effect between the seedling and jointing stages and across years (Table 1), indicating that the compensation effect varied with interannual climatic conditions, maize growth stage and mulch film type [21]. Across the two years, the compensation coefficients were higher at the seedling stage than at the jointing stage, suggesting that mulch-induced soil warming provided stronger compensation for the thermal requirement of maize during the seedling stage [7]. At this stage, maize seedlings have less developed roots and leaves and are more sensitive to fluctuations in shallow soil temperature. By increasing 0–10 cm soil temperature, film mulching promoted seedling growth and early canopy development [28]. Previous studies have shown that the warming effect of film mulching is usually most pronounced during early crop growth stages and gradually weakens or disappears as the canopy develops. Accordingly, the compensation effect of film mulching was weaker at the jointing stage than at the seedling stage [29,30].

In 2023, the compensation coefficients of the biodegradable film treatments were generally slightly higher than that of PM, and this difference became more pronounced in 2024. This pattern may be related to the diminishing marginal effect of soil temperature compensation. Although PM produced a greater increase in soil temperature, the additional soil heat accumulation was not converted proportionally into earlier maize development, resulting in a relatively lower compensation coefficient [7]. After maize entered the jointing stage, the compensation coefficients decreased in all treatments, which was related to the decline in the mulching effects caused by film degradation and canopy interception of solar radiation. In addition, the compensation coefficients were not fixed coefficients for a given mulching treatment, but stage-, year- and treatment-specific empirical coefficients. The interannual variation in compensation coefficients may be associated with differences in meteorological conditions, soil conditions, film degradation, and maize growth dynamics.

For daily compensation values, the overall pattern among mulching treatments was approximately PM = DM3 > DM2 > DM1, and the differences between the seedling and jointing stages were small. These results indicate that the compensation effect under film mulching was related to film type and thickness [31]. The warming effect of biodegradable films is usually weaker than that of PM, which is associated with the higher water vapor permeability of their film-forming materials and their degradation under field conditions [32]. For biodegradable films, thinner films generally have weaker warming capacity and tend to crack and degrade earlier, further reducing their ability to warm the soil [33,34].

These results indicate that early maize growth under film mulching is jointly regulated by air temperature and soil temperature accumulation. Therefore, the same GDDs_air_ do not necessarily indicate the same phenological progress in mulched and non-mulched treatments. Incorporating the compensation effect of film mulching into growing degree days calculation can help overcome the insufficient representation of early canopy development by GDDs_air_ in mulched treatments [35]. Moreover, the warming effect of film mulching changes as maize growth progresses, and heat requirements and temperature sensitivity differ among growth stages. A stage-specific compensation coefficient is therefore more suitable for quantifying the compensation effect at different growth stages [36].

### 2.2. Effects of Film Mulching on LAI Dynamics

**Figure 1:**
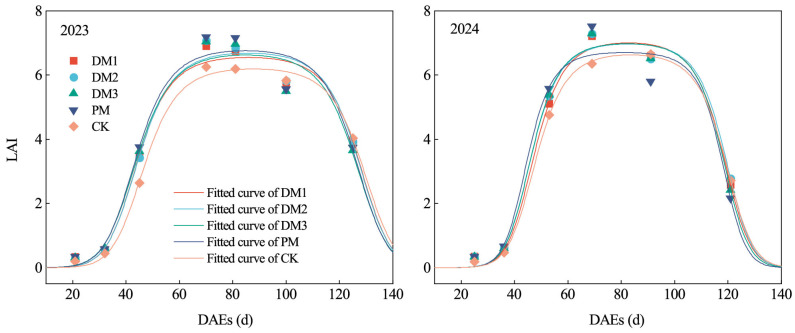
Modified Logistic fitting of maize leaf area index (LAI) dynamics against days after emergence (DAEs) under different treatments.

The modified Logistic model described changes in maize LAI with increasing DAEs well under different mulching treatments (Figure 1). The characteristic values of the fitted curves showed that mulching treatments affected not only LAI_max_ but also the timing of the maximum LAI growth rate and peak LAI (Table 2). The LAI_max_ values fitted by the modified Logistic model were higher under film mulching treatments than under CK.

Film mulching increased shallow soil temperature during early maize growth, thereby promoting early canopy development and advancing the timing of both the maximum LAI growth rate and peak LAI [37]. For example, in 2023, the maximum LAI growth rate occurred at 43, 43, 42 and 42 DAEs in DM1, DM2, DM3 and PM, respectively, which were 2–3 d earlier than in CK. In 2024, the corresponding timings were 46, 45, 45 and 44 DAEs, which were 1–3 d earlier than in CK. The maximum growth rate occurred later in DM1 and DM2 than in PM and DM3. This delay was associated with the weaker soil-warming capacity of thinner biodegradable films and the reduced soil-warming effect caused by their faster degradation [31,38].

The soil-warming effect and compensation effect of film mulching during the seedling and jointing stages were important drivers of earlier maize canopy development. Because the warming effects differed among film mulching treatments, the extent to which canopy development was advanced also varied, resulting in clear shifts in canopy developmental timing among treatments [22]. Correcting this timing shift can help improve the accuracy of unified simulation and more accurately describe LAI dynamics under film mulching.

### 2.3. Model Validation and Comparison

**Figure 2:**
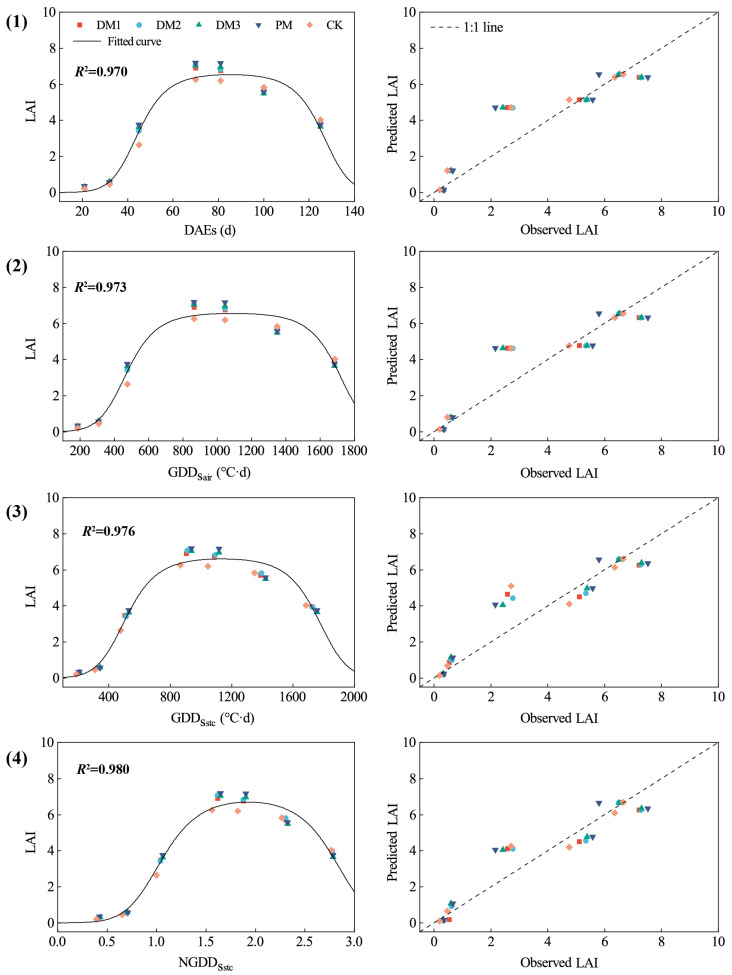
Calibration and independent validation of modified Logistic LAI models driven by different time scales. Rows (1)–(4) correspond to the DAEs-, GDDs_air_-, GDDs_stc_- and NGDDs_stc_-driven models, respectively. Left panels show fitted curves based on 2023 calibration data, and right panels show observed versus predicted LAI values for 2024 independent validation data. Calibration parameters and validation statistics are reported in Table 3.

Model performance was evaluated using the coefficient of determination (*R*^2^), root mean square error (RMSE), and Willmott’s index of agreement (*d*). The modified Logistic LAI models driven by different time scales all accurately described the unimodal pattern of LAI dynamics during the growing season (Figure 2). During model calibration, the models driven by DAEs, GDDs_air_, GDDs_stc_ and NGDDs_stc_ all showed good fitting performance, with *R*^2^ values of 0.970, 0.973, 0.976 and 0.980, respectively.

Independent validation showed that the DAEs- and GDDs_air_-driven models had similar predictive performance, with *R*^2^, RMSE and Willmott’s *d* values of 0.862, 1.011 and 0.962, and 0.870, 0.982 and 0.965, respectively (Table 3). Compared with DAEs, GDDs_air_ provided only limited improvement in model prediction. This suggests that a thermal time scale based only on air temperature cannot adequately reflect the advancement of early canopy development caused by film mulching. This is mainly because film mulching promotes early maize canopy development by increasing shallow soil temperature, whereas GDDs_air_ do not account for the compensation of this soil temperature increment for air temperature accumulation [39].

After the soil temperature compensation mechanism was introduced, model predictive performance improved. The GDDs_stc_-driven model had *R*^2^, RMSE and Willmott’s *d* values of 0.882, 0.935 and 0.967, respectively. Its RMSE values were 7.52% and 4.79% lower than those of the DAEs- and GDDs_air_-driven models, respectively. These results indicate that incorporating soil temperature compensation during the seedling and jointing stages into growing degree days calculation partly corrected the shift in canopy developmental timing under film mulching and improved model prediction reliability [22].

After GDDs_stc_ were segmentally normalized using the inflection and maximum points of the DAEs-LAI modified Logistic curves, NGDDs_stc_ mapped LAI dynamics across different years and film mulching treatments onto a unified relative developmental scale. This normalization helped reduce the influence of treatment-specific shifts in canopy developmental timing on model prediction. The unified developmental scale also helped reduce errors caused by differences in sampling dates and observation intervals among years. Among the four models, the NGDDs_stc_-driven model performed best, with *R*^2^, RMSE and Willmott’s *d* values of 0.906, 0.833 and 0.974, respectively. Its RMSE values were 17.61%, 15.17% and 10.91% lower than those of the DAEs-, GDDs_air_- and GDDs_stc_-driven models, respectively. In the 1:1 scatter plot of observed versus predicted values, points from the NGDDs_stc_-driven model were more closely distributed around the 1:1 line, indicating better predictive performance (Figure 2).

### 2.4. Implications and Limitations

This study shows that representing maize canopy development under film mulching using only DAEs or GDDs_air_ has limitations. Film mulching increased soil temperature during early maize growth, advancing the timing of the maximum LAI growth rate and peak LAI. These changes in early development were difficult to capture accurately using DAEs or GDDs_air_ alone. Therefore, incorporating the compensation effect of film mulching into thermal time calculation is important. GDDs_stc_ provided a more accurate thermal time scale for simulating LAI dynamics under film mulching. Compared with DAEs and GDDs_air_, GDDs_stc_ effectively corrected shifts in canopy developmental timing caused by mulch-induced soil warming. Further segmental normalization of GDDs_stc_ based on the inflection and maximum points of the DAEs-LAI modified Logistic curves helped reduce errors caused by interannual variation and improved cross-year predictive performance. Existing crop models, such as AquaCrop, DSSAT and DNDC, usually use different forms of canopy growth modules. These modules include processes or parameters such as canopy growth coefficient, canopy decline coefficient, leaf expansion, leaf senescence, biomass partitioning and specific leaf area [40,41,42]. Although their canopy growth functions differ from the modified Logistic model used in this study, most models still rely on GDDs_air_ or related thermal time indicators to represent crop phenology and canopy development [43]. Some models have considered the effects of film mulching on soil evaporation, soil temperature or surface energy transfer. However, the effect of mulch-induced soil warming on crop canopy development remains difficult to quantify accurately [44]. Zou et al. [21] suggested that compensation coefficients can provide an efficient way to incorporate the warming effect of film mulching into crop models. Ding et al. [7] also considered the development of a film mulching compensation module an important direction for crop model improvement. Therefore, GDDs_stc_ and NGDDs_stc_ are not limited to Logistic models but may also provide a useful reference for improving the canopy developmental time scale in different crop models.

Several limitations in this study should be noted. First, the model was developed and validated using only a two-year field experiment. Although NGDDs_stc_ showed good predictive performance in this study, their stability and transferability should be further tested using multi-year and multi-site datasets covering different cultivars, climatic conditions and mulch film types. Second, this study focused mainly on LAI dynamics and thermal time correction, while environmental stress factors, such as water stress, nutrient limitation and extreme temperature stress, were not incorporated into the model. Future studies could couple compensation coefficients, GDDs_stc_ or NGDDs_stc_ with phenology or canopy development modules in crop models such as AquaCrop, DSSAT and DNDC to evaluate their usefulness under broader environmental conditions. Finally, soil temperature was not monitored continuously on a daily basis. Some daily soil temperature data were estimated from periodic measurements using interpolation based on the relationship between air and soil temperature. This approach may not adequately capture short-term variation caused by weather fluctuations or rainfall events, which could affect the calculation of GDDs_stc_ and NGDDs_stc_. Therefore, future studies could use automatic soil temperature sensors and continuous monitoring to improve the accuracy of soil temperature compensation coefficients and the calculation of GDDs_stc_ and NGDDs_stc_.

## 3. Materials and Methods

### 3.1. Experimental Site and Design

**Figure 3:**
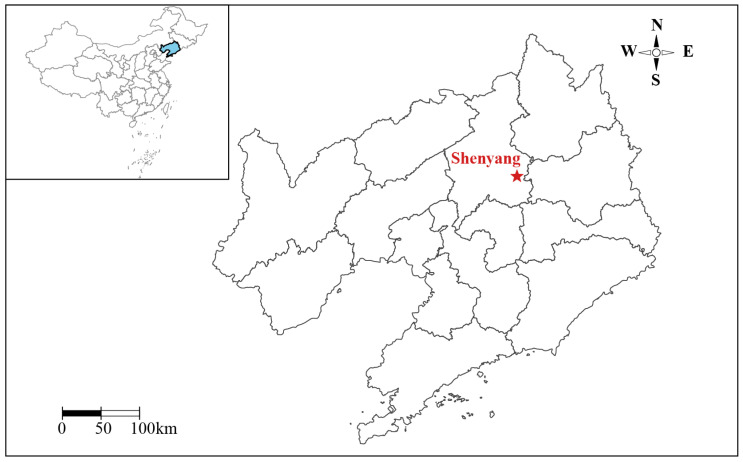
Geographic location of the experimental site in Shenyang, Liaoning Province, Northeast China.

Field experiments were conducted in 2023 and 2024 at the experimental station of Shenyang Agricultural University, Shenyang, Liaoning Province, China (Figure 3). The study area has a temperate continental monsoon climate, with a long-term mean annual temperature of 8.5 °C, a mean annual sunshine duration of 2743 h, and a mean annual precipitation of 716.2 mm, mainly concentrated from June to August. The soil at the experimental site is silty loam, with detailed properties provided in Table S1. Total precipitation during the maize seedling and jointing stages was 252.5 mm in 2023 and 226.9 mm in 2024 (Figure S1).

The maize cultivar used in this study was ‘Liangyu 99’ (Dandong Liangyu Seed Industry Co., Ltd., Dandong, China). Five treatments were established: biodegradable mulch films (Shanghai Hongrui Biotech Co., Ltd., Shanghai, China) with thicknesses of 0.006 mm (DM1), 0.008 mm (DM2) and 0.010 mm (DM3), a conventional plastic film (Xifeng Plastics (Group) Co., Ltd., Baishan, China) with a thickness of 0.010 mm (PM), and a no-mulching control (CK). The biodegradable mulch films were mainly composed of poly(butylene adipate-co-terephthalate) (PBAT, 70%) and polylactic acid (PLA, 10%). All mulch films were 1.2 m wide. The experiment followed a randomized block design with three replicates for each treatment. Each plot covered an area of 25.2 m^2^ (7 m × 3.6 m).

Maize was planted using a paired-row pattern, with a ridge width, ridge height and furrow width of 80 cm, 15 cm and 40 cm, respectively. The target planting density was 82,500 plants ha^−1^. Before sowing, controlled-release fertilizer (Chengdu Wintrue Holding Co., Ltd., Chengdu, China; N, P_2_O_5_, K_2_O ratio of 27:12:12) was applied at 750 kg ha^−1^, with no additional topdressing during the growing season. No irrigation was applied during the experiment. The sowing dates were 1 May 2023 and 27 April 2024, and the harvest dates were 27 September 2023 and 22 September 2024.

### 3.2. Sampling and Measurements

#### 3.2.1. Film Degradation

Three fixed observation areas were established in each plot. From the day of mulching, photographs were taken every 30 d with a camera positioned 0.5 m directly above each observation area to assess the degree of film degradation (Table S2). Film degradation was classified into six grades from 0 to 5 [45]. Grade 0 indicated no visible degradation; grade 1 indicated the initial appearance of cracks on the film surface; grade 2 indicated cracks of 2–5 cm; grade 3 indicated cracks larger than 5 cm; grade 4 indicated a uniform reticular cracking pattern with no large film fragments remaining on the soil surface; and grade 5 indicated that the film had almost disappeared, with no visible film or only minute fragments remaining.

#### 3.2.2. Soil Temperature

Soil temperature was measured using soil thermometers (Tianjin Jixing Instrument Factory, Tianjin, China) at depths of 5 and 10 cm. Measurements were taken at 07:00, 14:00 and 18:00 on each observation day, with an observation interval of 7 d. The mean of the 5 and 10 cm measurements was used to represent 0–10 cm soil temperature, and the mean of the three daily measurements was used as daily mean soil temperature (Figure S2). Because soil temperature was measured intermittently, daily soil temperature between two adjacent observation dates was estimated. A piecewise linear interpolation method corrected by air temperature was used. Specifically, the difference between soil temperature and air temperature was first linearly interpolated between adjacent observation dates, and the interpolated difference was then added back to the air temperature on the corresponding date. The detailed calculation procedure is shown in Equations (S1)–(S4).

#### 3.2.3. Leaf Area Index

Leaf area was measured several times during the maize growing season to capture canopy growth, peak formation and late-season decline (Figure S3). At each sampling, three representative maize plants were selected from each plot. The length and maximum width of all leaves were measured using a measuring tape, and LAI was calculated as follows [46]:

(1) $$\mathrm{LAI}=0.75\rho \frac{\sum_{i=1}^{m}\sum_{j=1}^{n}L_{ij}\times W_{ij}}{m}\times 10^{-8}$$

where *ρ* is planting density (plants ha^−1^); *m* is number of sampled plants; *n* is number of leaves; *L_ij_* is leaf length (cm); and *W_ij_* is maximum leaf width (cm). The factor 10^−8^ was used to convert leaf area per unit ground area from cm^2^ ha^−1^ to m^2^ m^−2^.

### 3.3. Calculation of Compensation Effect

Previous studies have shown that the soil-warming effect of film mulching is mainly concentrated during early maize growth, particularly at the seedling and jointing stages, and is most evident in the 0–10 cm soil layer [24,39]. Therefore, in this study, the compensation effect was calculated separately using 0–10 cm soil temperature during the seedling and jointing stages, and the compensation effect after the jointing stage was set to zero. In addition, CK was used as the baseline treatment, for which the compensation coefficient was also set to zero. The compensation coefficient was calculated as follows [7]:

(2) $$K=\frac{{\mathrm{GDDs}}_{\text{air-NM}}-{\mathrm{GDDs}}_{\text{air-FM}}}{{\mathrm{GDDs}}_{\text{soil-FM}}-{\mathrm{GDDs}}_{\text{soil-NM}}}$$

where *K* is the compensation coefficient; GDDs_air-NM_ is the air-temperature-based growing degree days of CK during the seedling or jointing stage (°C d); GDDs_air-FM_ is the air-temperature-based growing degree days of the film mulching treatments during the seedling or jointing stage (°C d); GDDs_soil-FM_ is the soil-temperature-based growing degree days of the film mulching treatments during the seedling or jointing stage (°C d); and GDDs_soil-NM_ is the soil-temperature-based growing degree days of CK over the same period when the film mulching treatments reached the corresponding phenological stage (°C d). The definitions of the seedling and jointing stages are shown in Table S3.

GDDs_air_ and GDDs_soil_ were calculated as follows [21]:

(3) $${\mathrm{GDDs}}_{\mathrm{air}}=\sum_{i=1}^{n}\max(T_{i\text{-}a}-T_{a,l},0)$$

(4) $${\mathrm{GDDs}}_{\mathrm{soil}}=\sum_{i=1}^{n}\max(T_{i\text{-}s}-T_{s,l},0)$$

where GDDs_air_ and GDDs_soil_ are growing degree days based on air temperature and soil temperature, respectively (°C d); *T_i-a_* and *T_i-s_* are the mean air temperature and mean soil temperature on day *i*, respectively (°C); *n* is the number of maize growth days (d); and *T_a,l_* and *T_s,l_* are the lower biological threshold temperatures for air and soil temperature, respectively, both set to 10 °C [47]. In this study, both mean air temperature and mean soil temperature were above their lower threshold temperatures. It should be noted that the increase in soil temperature caused by film mulching is not always beneficial for crop growth. Previous studies suggest that the suitable soil temperature range for maize growth is 25–35 °C [7]. In this study, soil temperature did not exceed the upper limit of this suitable range.

In general, the compensation effect of film mulching is related to the ratio between air temperature and soil temperature. A larger ratio indicates a greater compensation effect, and vice versa. The daily compensation value was calculated as follows:

(5) $$\Delta T=\frac{K\times (T_{s\text{-}FM}-T_{s\text{-}NM})(T_{a}-T_{a,l})}{\left(T_{s\text{-}NM}-T_{s,l}\right)}$$

where Δ*T* is the daily compensation value of the soil temperature increment under film mulching for air temperature (°C); *T_s-FM_* is the mean 0–10 cm soil temperature of the film mulching treatments (°C); *T_s-NM_* is the mean 0–10 cm soil temperature of CK (°C); and *T_a_* is the mean air temperature (°C).

The soil-temperature-compensated thermal time for each treatment was calculated as follows, with the compensation value set to zero for CK:

(6) $${\mathrm{GDDs}}_{\mathrm{stc}}={\mathrm{GDDs}}_{\mathrm{air}}+\sum_{i=1}^{n}\Delta T_{i}$$

where GDDs_stc_ are the soil-temperature-compensated growing degree days (°C d); *n* is the number of maize growth days (d); Δ*T_i_* is the compensation value on day *i* (°C).

### 3.4. Logistic Model

#### 3.4.1. Modified Logistic Model

Based on previous studies, the Logistic model was modified to describe LAI dynamics [26]. The model was expressed as follows:

(7) $$\mathrm{LAI}=\frac{a}{1+e^{\left(b+ct+dt^{2}\right)}}$$

where *t* is the time-scale variable, and *a*, *b*, *c* and *d* are parameters to be estimated.

#### 3.4.2. Data Normalization

**Figure 4:**
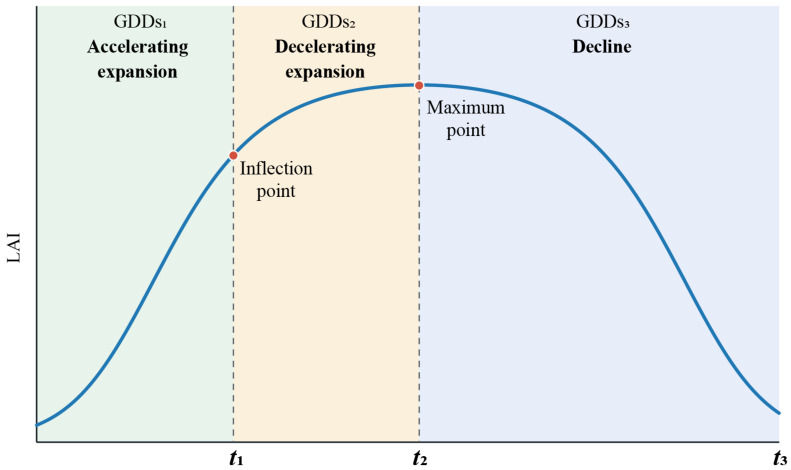
Schematic representation of maize LAI dynamics and growing degree days (GDDs) segmentation based on the modified Logistic model.

Maize LAI dynamics can differ markedly among years because of interannual climatic variation. In addition, differences in sampling dates and observation intervals among years may also affect model accuracy. To improve model performance, the modeling procedure was optimized in two steps. First, the modified Logistic model was used to fit the relationship between LAI and DAEs for each treatment (Figure 1). The inflection point, maximum point and their corresponding dates were then calculated, and LAI dynamics were divided into three stages using the inflection and maximum points as boundaries (Figure 4). Second, GDDs_stc_ were normalized for each treatment according to the redefined LAI developmental stages to obtain normalized soil-temperature-compensated growing degree days (NGDDs_stc_). This normalization was used to help reduce the influence of differences in heat accumulation among years and treatments on model accuracy.

The accumulated thermal time for each stage of LAI dynamics was calculated as follows:

(8) $$\left\{\begin{array}{l} {\mathrm{GDDs}}_{1}=\sum_{i=1}^{t_{1}}\left(T_{i\text{-}a}-T_{a,l}+\Delta T_{i}\right)\\ {\mathrm{GDDs}}_{2}=\sum_{i=t_{1}}^{t_{2}}\left(T_{i\text{-}a}-T_{a,l}+\Delta T_{i}\right)\\ {\mathrm{GDDs}}_{3}=\sum_{i=t_{2}}^{t_{3}}\left(T_{i\text{-}a}-T_{a,l}+\Delta T_{i}\right) \end{array}\right.$$

where GDDs_1_ is the accumulated thermal time during the period when LAI increased with a positive growth rate (°C d); GDDs_2_ is the accumulated thermal time during the period when LAI continued to increase but its growth rate declined (°C d); GDDs_3_ is the accumulated thermal time during the period when LAI decreased (°C d); *t*_1_ is the DAEs corresponding to the inflection point of the modified Logistic curve (d); *t*_2_ is the DAEs corresponding to the maximum point of the modified Logistic curve (d); and *t*_3_ is the DAEs corresponding to maize maturity (d).

NGDDs_stc_ were calculated as follows:

(9) $${\mathrm{NGDDs}}_{\mathrm{stc}}=\left\{\begin{array}{c} \frac{{\mathrm{GDDs}}_{\text{stc-k}}}{{\mathrm{GDDs}}_{1}}\ldots \ldots \ldots \ldots \ldots \ldots \ldots \ldots \ldots \ldots \ldots 0<t_{k}\leq t_{1}\\ 1+\frac{{\mathrm{GDDs}}_{\text{stc-k}}-{\mathrm{GDDs}}_{1}}{{\mathrm{GDDs}}_{2}}\ldots \ldots \ldots \ldots \ldots \ldots t_{1}<t_{k}\leq t_{2}\\ 2+\frac{{\mathrm{GDDs}}_{\text{stc-k}}-{\mathrm{GDDs}}_{1}-{\mathrm{GDDs}}_{2}}{{\mathrm{GDDs}}_{3}}\ldots \ldots t_{2}<t_{k}\leq t_{3} \end{array}\right.$$

where *t_k_* is the DAEs corresponding to the *k*-th LAI observation and GDDs_stc-k_ are the soil-temperature-compensated growing degree days corresponding to the *k*-th LAI observation date (°C d). To ensure the independence of model validation, the segmentation boundaries for NGDDs_stc_ in 2024 were determined from the relative positions of the inflection and maximum points in total GDDs_stc_ in 2023. The data from 2024 were used only for independent model validation, without any LAI data from 2024 being used to estimate model parameters or NGDDs_stc_ segmentation boundaries.

### 3.5. Model Calibration and Validation

Modified Logistic LAI models were constructed separately using DAEs, GDDs_air_, GDDs_stc_ and NGDDs_stc_ as independent variables (Table S4). All observations from the five treatments in 2023 were pooled for model calibration, and parameters were estimated by nonlinear least-squares regression in Python 3.14.5, with the least_squares function from the scipy.optimize module of SciPy. The observations in 2024 were used for independent validation (Table S5).

In terms of operational availability, the fitted model parameters and the NGDDs_stc_ segmentation rule were obtained from the 2023 calibration dataset. The stage-specific compensation coefficients were calculated separately for each year and treatment using the corresponding air temperature, soil temperature and growth stage information. Air temperature, soil temperature and growth stage information can be obtained from field monitoring during the target year and used to calculate GDDs_air_ and GDDs_stc_.

### 3.6. Statistical Analysis

Data preprocessing was performed using Microsoft Excel 365 (Microsoft Corp., Redmond, WA, USA). Figures were generated using Origin 2021 software (OriginLab Corp., Northampton, MA, USA).

Model performance was evaluated using the coefficient of determination (*R*^2^), root mean square error (RMSE) and Willmott’s index of agreement (*d*). *R*^2^ was used to describe the proportion of variation in the observed values explained by the model. Higher *R*^2^ values indicate a better model fit. RMSE was used to quantify the magnitude of simulation error, with smaller values indicating higher predictive accuracy. Willmott’s *d* was used to evaluate the agreement between predicted and observed values. It generally ranges from 0 to 1, with values closer to 1 indicating better model performance. These metrics were calculated as follows:

(10) $$R^{2}=1-\frac{\sum_{i=1}^{n}{\left(O_{i}-S_{i}\right)}^{2}}{\sum_{i=1}^{n}{\left(O_{i}-\bar{O}\right)}^{2}}$$

(11) $$\mathrm{RMSE}=\sqrt{\frac{\sum_{i=1}^{n}{\left(O_{i}-S_{i}\right)}^{2}}{n}}$$

(12) $$d=1-\frac{\sum_{i=1}^{n}{\left(O_{i}-S_{i}\right)}^{2}}{\sum_{i=1}^{n}{\left(\left|S_{i}-\bar{O}\right|+\left|O_{i}-\bar{O}\right|\right)}^{2}}$$

where *O_i_* is the observed value, *S_i_* is the predicted value, $\bar{O}$ is the mean observed value, and *n* is the sample size.

## 4. Conclusions

This study quantified the compensation of increased soil temperature for GDDs_air_ during early maize growth under different film mulching treatments. The results showed that film-induced increases in shallow soil temperature partly compensated for air-temperature-based thermal accumulation. The compensation effect was stronger at the seedling stage than at the jointing stage and differed among mulching treatments, indicating the limitations of GDDs_air_ in simulating maize LAI dynamics under film mulching conditions.

Compared with growing degree days calculated only from air temperature, incorporating the compensation effect into growing degree days calculation enabled the model to better correct the shift in canopy developmental timing caused by film mulching and improved predictive performance. After segmental normalization of GDDs_stc_ based on the characteristic points of the fitted LAI curves, model performance was further improved, with *R*^2^, RMSE and Willmott’s *d* values of 0.906, 0.833 and 0.974, respectively. These results indicate the importance of the mulching-induced compensation effect in canopy development simulation. GDDs_stc_ and their normalized form, NGDDs_stc_, provide an interpretable thermal time correction for simulating maize canopy development under biodegradable and conventional plastic film mulching, and may serve as a reference for improving canopy development modules in crop models.

## Figures and Tables

**Table 1:** Stage-specific soil temperature compensation coefficients and daily compensation values under different mulching treatments.

Year	Treatment	Compensation Coefficient		Daily Compensation Value (°C)	
		Seedling Stage	Jointing Stage	Seedling Stage	Jointing Stage
2023	DM1	1.00	0.57	0.59	0.63
	DM2	1.07	0.49	0.81	0.58
	DM3	0.99	0.77	1.06	1.28
	PM	0.92	0.64	1.07	1.29
2024	DM1	0.86	0.61	0.54	0.66
	DM2	0.84	0.43	0.86	0.79
	DM3	0.80	0.75	1.24	1.44
	PM	0.59	0.52	1.18	1.42

Note: DM1, DM2 and DM3 represent biodegradable films with thicknesses of 0.006, 0.008 and 0.010 mm, respectively; and PM, conventional plastic film. Values were calculated using air temperature and 0–10 cm soil temperature averaged across the three replicates for each treatment. Because these values were derived from treatment-level mean temperature data rather than calculated separately for each replicate, no separate statistical analysis was conducted.

**Table 2:** Characteristic points of the modified Logistic LAI models under different treatments.

Year	Treatment	LAI_max_	DAEs of Inflection Points (d)	DAEs of Maximum Points (d)
2023	DM1	6.55	43	86
	DM2	6.69	43	86
	DM3	6.63	42	85
	PM	6.76	42	85
	CK	6.20	45	88
2024	DM1	7.00	46	83
	DM2	6.97	45	83
	DM3	6.98	45	82
	PM	6.74	44	81
	CK	6.63	47	83

Note: DAEs, days after emergence. Values were obtained from modified Logistic curves shown in Figure 1. Because these values were derived from treatment-level mean LAI rather than calculated separately for each replicate, no separate statistical analysis was conducted.

**Table 3:** Calibration parameters and independent validation performance of modified Logistic LAI models driven by different time scales.

Time Scale	Model	Calibration Parameters				Validation Statistics		
		*a*	*b*	*c*	*d*	*R* ^2^	RMSE	Willmott’s *d*
DAEs	DAEs-LAI	6.8437	10.7467	−0.323809	0.001888	0.862	1.011	0.962
GDDs_air_	GDDs_air_-LAI	6.7634	7.5036	−0.020132	9.22 × 10^−6^	0.870	0.982	0.965
GDDs_stc_	GDDs_stc_-LAI	6.9537	7.0558	−0.017591	7.75 × 10^−6^	0.882	0.935	0.967
NGDDs_stc_	NGDDs_stc_-LAI	7.6600	8.3244	−10.663191	2.768422	0.906	0.833	0.974

Note: The modified Logistic model was expressed as LAI = *a*/[1 + exp(*b* + *ct* + *dt*^2^)], where *t* is the corresponding time scale. GDDs_air_, air-temperature-based growing degree days; GDDs_stc_, soil-temperature-compensated growing degree days; NGDDs_stc_, normalized GDDs_stc_; RMSE, root mean square error. Parameter *a* represents the upper scale of LAI; *b* determines the curve position; *c* controls the early increasing trend; and *d* is a quadratic term that enables the fitted curve to decline during the late growing season after LAI reaches its peak.

## Data Availability

The original contributions presented in this study are included in the article and Supplementary Materials. Further inquiries can be directed to the corresponding author.

## Supplementary Materials

The following supporting information can be downloaded at: https://www.mdpi.com/article/10.3390/plants15142163/s1, Figure S1: Air temperature and precipitation during the maize growing seasons in 2023 and 2024; Figure S2: Temporal dynamics of mean 0–10 cm soil temperature under different treatments during the maize seedling and jointing stages; Figure S3: Temporal dynamics of maize leaf area index (LAI) under different treatments; Table S1: Soil properties at a 0–40 cm depth before the experiment; Table S2: Degradation process of films; Table S3: Dates of maize seedling and jointing stages under different treatments; Table S4: Observed maize LAI and corresponding time-scale variables used for model calibration; Table S5: Observed maize LAI and corresponding time-scale variables used for independent model validation.

## Abbreviations

The following abbreviations are used in this manuscript:

DM1	Biodegradable mulch film with a thickness of 0.006 mm
DM2	Biodegradable mulch film with a thickness of 0.008 mm
DM3	Biodegradable mulch film with a thickness of 0.010 mm
PM	Conventional plastic film
CK	No-mulching control
DAEs	Days after emergence
GDDs_air_	Air-temperature-based growing degree days
GDDs_stc_	Soil-temperature-compensated growing degree days
NGDDs_stc_	Normalized soil-temperature-compensated growing degree days
LAI	Leaf area index
PBAT	Poly(butylene adipate-co-terephthalate)
PLA	Polylactic acid
PE	Polyethylene
