# Supplementary material for: Soil-Temperature-Compensated Growing Degree Days Improve Unified Simulation of Maize LAI Dynamics Across Film Mulching Treatments
Source: Plants (Basel). 2026 Jul 14;15(14):2163. doi: 10.3390/plants15142163 (PMC13414788; doi:10.3390/plants15142163)
Supplement: Supplementary file 1 [file plants-15-02163-s001.zip › plants-4412553-supplementary.pdf]

plants

MDPI

Supplementary Materials

Soil-Temperature-Compensated Growing Degree Days Improve Unified Simulation of Maize LAI Dynamics Across Film Mulching Treatments

Content

Figure S1. Air temperature and precipitation during the maize growing seasons in 2023 and 2024.

Figure S2. Temporal dynamics of mean 0–10-cm soil temperature under different treatments during the maize seedling and jointing stages.

Figure S3. Temporal dynamics of maize leaf area index (LAI) under different treatments.

Table S1. Soil properties at a 0–40 cm depth before the experiment.

Table S2. Degradation process of films.

Table S3. Dates of maize seedling and jointing stages under different treatments.

Table S4. Observed maize LAI and corresponding time-scale variables used for model calibration.

Table S5. Observed maize LAI and corresponding time-scale variables used for independent model validation.

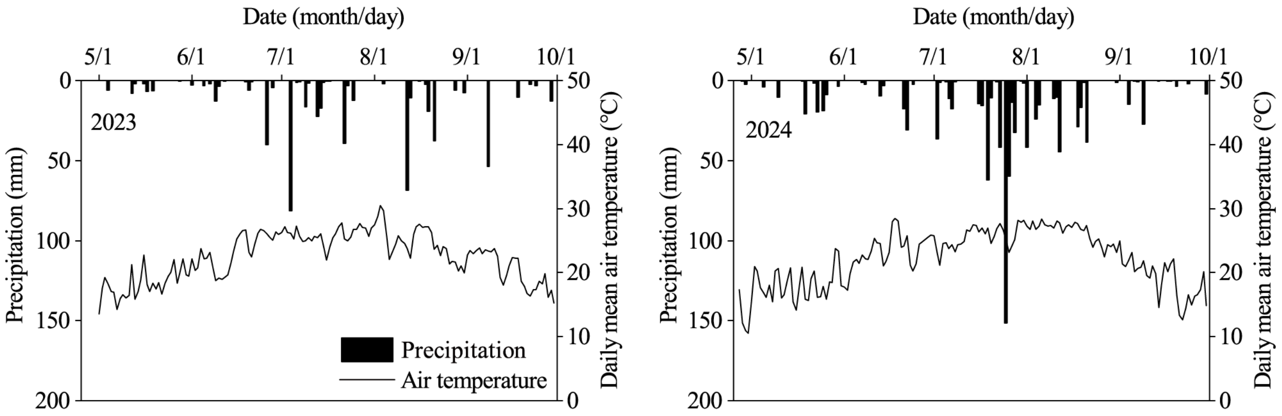

Figure S1. Air temperature and precipitation during the maize growing seasons in 2023 and 2024.

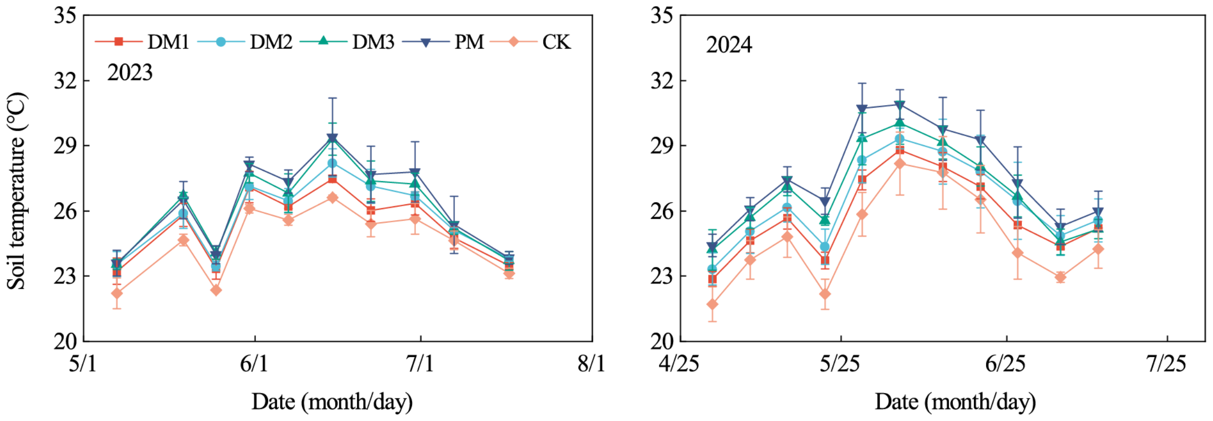

Figure S2. Temporal dynamics of mean 0–10-cm soil temperature under different treatments during the maize seedling and jointing stages. DM1, DM2 and DM3 represent biodegradable films with thicknesses of 0.006, 0.008 and 0.010 mm, respectively; PM, conventional plastic film; and CK, no-mulching control. Error bars show standard errors of the mean (n = 3).

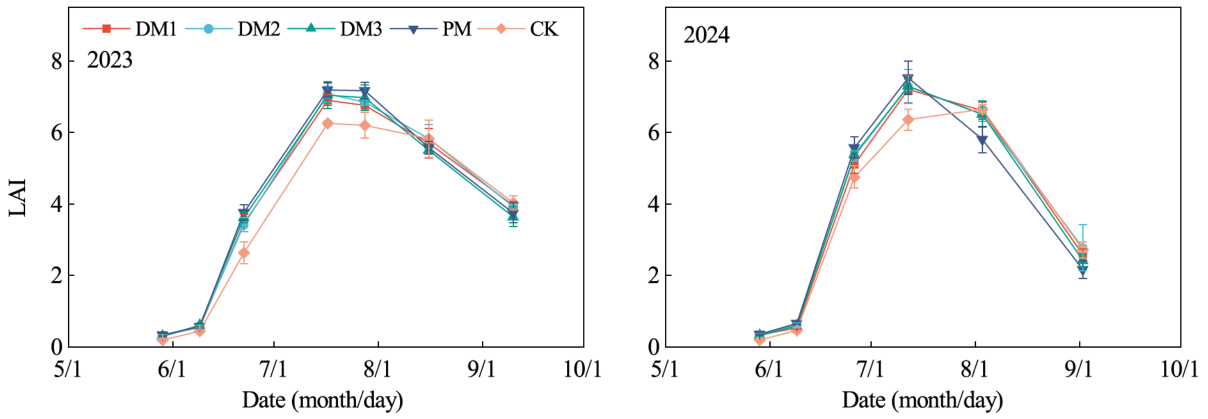

Figure S3. Temporal dynamics of maize leaf area index (LAI) under different treatments. Error bars show standard errors of the mean (n = 3).

https://doi.org/10.3390/plants15142163

Table S1. Soil properties at a 0–40 cm depth before the experiment.

BD (g cm^{-3})	SOM (g kg^{-1})	TN (g kg^{-1})	AP (mg kg^{-1})	AK (mg kg^{-1})	pH
1.34	20.8	0.87	8.9	75.6	7.28

Note: BD, bulk density; SOM, soil organic matter; TN, total nitrogen; AP, available phosphorus; AK, available potassium.

Table S2. Degradation process of films.

Year	Treatment	Days after mulching					
		0	30	60	90	120	150
2023	DM1	0	0	2	3	4	4
	DM2	0	0	1	2	3	4
	DM3	0	0	0	2	3	4
	PM	0	0	0	0	0	0
2024	DM1	0	0	3	5	5	5
	DM2	0	0	0	4	5	5
	DM3	0	0	0	4	5	5
	PM	0	0	0	0	0	0

Note: Film degradation was assessed visually using a 0–5 scale: 0, no visible degradation; 1, initial surface cracking; 2, cracks of 2–5 cm; 3, cracks >5 cm; 4, uniform net-like cracking with no large residual fragments on the soil surface; and 5, near-complete film disappearance, with only invisible or minute residues remaining.

Table S3. Dates of maize seedling and jointing stages under different treatments.

Year	Treatment	Seedling period	Jointing period
2023	DM1	1 May–16 June	17 June–11 July
	DM2	1 May–16 June	17 June–10 July
	DM3	1 May–15 June	16 June–8 July
	PM	1 May–15 June	16 June–8 July
	CK	1 May–20 June	21 June–15 July
2024	DM1	27 April–14 June	15 June–8 July
	DM2	27 April–13 June	14 June–6 July
	DM3	27 April–13 June	14 June–5 July
	PM	27 April–12 June	13 June–4 July
	CK	27 April–17 June	18 June–12 July

Table S4. Observed maize LAI and corresponding time-scale variables used for model calibration.

Year	Treatment	DAEs (d)	$GDDs_{air}$ (°C d)	$GDDs_{stc}$ (°C d)	$NGDDs_{stc}$	LAI
2023	DM1	21	186.60	200.22	0.4205	0.3300
		32	309.50	329.31	0.6916	0.5624
		45	475.90	504.44	1.0407	3.4656
		70	865.85	905.01	1.6169	6.9094
		81	1046.30	1085.46	1.8765	6.7543
		100	1349.65	1388.81	2.3062	5.7011
		125	1684.70	1723.86	2.7778	3.9598
	DM2	21	186.60	203.45	0.4192	0.3109
		32	309.50	334.61	0.6895	0.5770
		45	475.90	513.70	1.0410	3.4235
		70	865.85	912.25	1.6159	7.0796
		81	1046.30	1092.70	1.8762	6.8402
		100	1349.65	1396.05	2.3062	5.8239
		125	1684.70	1731.10	2.7778	3.9287
	DM3	21	186.60	209.19	0.4299	0.3041
		32	309.50	343.53	0.7059	0.6046
		45	475.90	530.89	1.0633	3.6265
		70	865.85	937.49	1.6455	7.0446
		81	1046.30	1117.94	1.9039	6.9710
		100	1349.65	1421.29	2.3240	5.4965
		125	1684.70	1756.34	2.7835	3.6461
	PM	21	186.60	207.02	0.4254	0.3441
		32	309.50	343.96	0.7067	0.5817
		45	475.90	530.71	1.0629	3.7656
		70	865.85	938.39	1.6460	7.1897
		81	1046.30	1118.84	1.9040	7.1698
		100	1349.65	1422.19	2.3240	5.5702
		125	1684.70	1757.24	2.7835	3.7521
	CK	21	186.60	186.60	0.3921	0.1955
		32	309.50	309.50	0.6503	0.4440
		45	475.90	475.90	1.0000	2.6360
		70	865.85	865.85	1.5600	6.2522
		81	1046.30	1046.30	1.8191	6.1960
		100	1349.65	1349.65	2.2646	5.8301
		125	1684.70	1684.70	2.7645	4.0344

Note: DAEs, days after emergence; $GDDs_{air}$, air-temperature-based growing degree days; $GDDs_{stc}$, soil-temperature-compensated growing degree days; $NGDDs_{stc}$, normalized $GDDs_{stc}$.

Table S5. Observed maize LAI and corresponding time-scale variables used for independent model validation.

Year	Treatment	DAEs (d)	$GDDs_{air}$ (°C d)	$GDDs_{stc}$ (°C d)	$NGDDs_{stc}$	LAI
2024	DM1	25	202.40	218.88	0.4648	0.3450
		36	320.25	342.31	0.4979	0.5414
		53	559.60	587.36	1.1694	5.1085
		69	791.60	829.23	1.5213	7.2106
		91	1154.95	1192.58	2.0488	6.6077
		121	1634.15	1671.78	2.7309	2.5809
	DM2	25	202.40	226.66	0.4700	0.3424
		36	320.25	354.22	0.7345	0.6279
		53	559.60	603.55	1.1761	5.3400
		69	791.60	845.55	1.5273	7.2888
		91	1154.95	1208.90	2.0534	6.5029
		121	1634.15	1688.10	2.7322	2.7793
	DM3	25	202.40	237.21	0.4898	0.3313
		36	320.25	368.84	0.7617	0.5988
		53	559.60	626.93	1.2053	5.3854
		69	791.60	873.14	1.5596	7.2944
		91	1154.95	1236.49	2.0790	6.5112
		121	1634.15	1715.69	2.7394	2.4217
	PM	25	202.40	234.68	0.4853	0.3518
		36	320.25	367.00	0.7589	0.6692
		53	559.60	626.31	1.2055	5.5821
		69	791.60	871.17	1.5579	7.5260
		91	1154.95	1234.52	2.0776	5.8011
		121	1634.15	1713.72	2.7390	2.1672
	CK	25	202.40	202.40	0.4298	0.1917
		36	320.25	320.25	0.6801	0.4720
		53	559.60	559.60	1.1287	4.7545
		69	791.60	791.60	1.4654	6.3551
		91	1154.95	1154.95	1.9927	6.6570
		121	1634.15	1634.15	2.7149	2.7166

https://doi.org/10.3390/plants15142163

A piecewise linear interpolation method corrected by air temperature was used to estimate daily soil temperature, which was calculated as follows:

$$\Delta T_i = T_{s,i} - T_{a,i} \tag{S1}$$

$$\Delta T_{i+1} = T_{s,i+1} - T_{a,i+1} \tag{S2}$$

$$\Delta T(\tau) = \Delta T_i + \frac{\Delta T_{i+1} - \Delta T_i}{\tau_{i+1} - \tau_i}(\tau - \tau_i) \tag{S3}$$

$$T_s(\tau) = T_a(\tau) + \Delta T(\tau) \tag{S4}$$

where τ_i and τ_{i+1} are two adjacent soil-temperature observation dates, and τ is any date between τ_i and τ_{i+1}; $T_{s,i}$ and $T_{s,i+1}$ are the observed soil temperatures on days τ_i and τ_{i+1}, respectively; $T_{a,i}$, $T_{a,i+1}$, and $T_a(\tau)$ are the corresponding air temperatures on days τ_i, τ_{i+1} and τ, respectively; ΔT_i and ΔT_{i+1} are the observed differences between soil temperature and air temperature on days τ_i and τ_{i+1}, respectively; $\Delta T(\tau)$ is the interpolated difference between soil temperature and air temperature on day τ; and $T_s(\tau)$ is the estimated daily soil temperature on day τ.

https://doi.org/10.3390/plants15142163
